# Serum levels of galactose-deficient immunoglobulin (Ig) A1 and related immune complex are associated with disease activity of IgA nephropathy

**DOI:** 10.1007/s10157-013-0921-6

**Published:** 2014-01-30

**Authors:** Yusuke Suzuki, Keiichi Matsuzaki, Hitoshi Suzuki, Keiko Okazaki, Hiroyuki Yanagawa, Norio Ieiri, Mitsuhiro Sato, Toshinobu Sato, Yoshio Taguma, Joe Matsuoka, Satoshi Horikoshi, Jan Novak, Osamu Hotta, Yasuhiko Tomino

**Affiliations:** 1 Division of Nephrology, Department of Internal Medicine, Juntendo University Faculty of Medicine, Hongo 2-1-1, Bunkyo-ku, Tokyo, Japan; 2 Department of Nephrology, Sendai Shakaihoken Hospital, Tutumi-cho 3-16-1, Aoba-ku, Sendai, Miyagi Japan; 3 Hotta Osamu Clinic, Rokuchonome Minami-cho 2-39, Wakabayashi-ku, Sendai, Miyagi Japan; 4 Faculty of Medicine, Clinical Research Center, Juntendo University, Hongo 2-1-1, Bunkyo-ku, Tokyo, Japan; 5 Department of Microbiology, University of Alabama at Birmingham, Birmingham, AL USA

**Keywords:** IgA nephropathy, Disease activity, Underglycosylated IgA, Immune complex, Biomarker

## Abstract

**Background:**

The primary abnormal manifestation in immunoglobulin A nephropathy (IgAN) is recurring bouts of hematuria with or without proteinuria. Although immunohistochemical analysis of renal biopsy tissue remains the gold standard not only for diagnosis but also for evaluating the activity of IgAN, new sensitive and reasonably specific noninvasive tests are emerging to guide therapeutic strategy applicable to all stages of IgAN. The present study examined serum levels of galactose-deficient IgA1 (Gd-IgA1) and its immune complex (IgA/IgG-IC) as noninvasive markers for the disease activity.

**Methods:**

We enrolled 50 IgAN patients (male 40 %, median age 37 years) showing complete or partial clinical remission after steroid pulse therapy with tonsillectomy (TSP) whose clinical data and serum could be followed up for 3–5 years.

**Results:**

Cross-sectional analysis revealed that the degree of hematuria and proteinuria were significantly associated with levels of Gd-IgA1 and levels of IgA/IgG-IC. Longitudinal analysis further showed that from the group of 44 patients with heavy hematuria before TSP, 31 patients showed complete disappearance of hematuria (group A), but the remaining patients did not (group B). Although the levels of Gd-IgA1 and IgA/IgG-IC in the two groups before TSP were similar, percentage decrease of Gd-IgA1 and IgA/IgG-IC levels in group A was significantly higher than in group B.

**Conclusion:**

Disease activity of IgAN assessed by hematuria and proteinuria correlated with serum levels and changes of Gd-IgA1 and IgA/IgG-IC. These new noninvasive disease activity markers can be useful for future activity scoring system and guiding therapeutic approaches.

## Introduction

The primary abnormal manifestation of immunoglobulin A nephropathy (IgAN) is recurring bouts of hematuria with or without proteinuria. However, IgAN has a disease spectrum with many common manifestations, where mesangial IgA immune deposits instigate glomerular damage via unknown mechanisms [1]. From clinical practice, it is known that approximately 30–40 % of IgAN patients progress to end-stage kidney disease within 20 years [1, 2], whereas 10–20 % of patients show spontaneous clinical remission [1–5]. However, there is no definitive method for discriminating patients with these different outcomes. Thus, the highly variable clinical course and unpredictable progression of IgAN hinder its treatment strategy.

Urinary protein levels may provide acceptable indicators of prognosis [1, 6–10]. However, assessing IgAN activity based on proteinuria should be carefully considered because proteinuria may partly be due to secondary focal segmental glomerulosclerosis (FSGS), known as ‘burned-out IgAN’, depending on the timing of biopsy during the clinical course [9]. Hematuria is the most important indicator of IgAN activity [1, 6, 7], but clinical evaluation using hematuria can be problematic because there are limitations to its quantification because of false-positive/negative reactions in dipstick tests. The clinical detection of urinary casts and dysmorphic red blood cells accompanying either macroscopic or microscopic hematuria clearly indicate that urinary tract bleeding is glomerular in origin, but they do not accurately indicate disease activity.

Immunohistochemical analysis of renal biopsy specimens is the gold standard for diagnosing and evaluating IgAN activity. However, over the prolonged clinical course of IgAN (approximately 20 years) the histological phenotype is dependent on the timing of renal biopsy [11]. In many countries, abnormalities found during urinalysis may be overlooked or purposely not followed up by further examination until renal function impairment is evident [6]. This raises a controversial issue among nephrologists of whether to perform renal biopsy in circumstances without renal function impairment or nephritic range proteinuria because of a perception that a specific treatment is not yet available. Routine screening for urinary abnormalities is performed for all school-aged children in Japan [5, 12, 13]. Furthermore, symptom-free individuals with microscopic hematuria are more likely to undergo renal biopsy, leading to increased diagnosis of IgAN in Japan. However, it is a common practice not to recommend renal biopsy for patients presenting with isolated hematuria or mild proteinuria in the UK, Canada, and the USA, where renal biopsy is reserved for those who develop increasing proteinuria or worsening renal function [6]. Differences in the pathological variables used for renal prognosis in the Japanese and Oxford classifications may partly account for the timing of renal biopsy [14, 15]. Renal biopsies cannot be performed frequently because of the risks involved with the procedure and for socioeconomic reasons. Therefore, renal biopsy is still a snapshot evaluation method and is not a practical method for determining disease activity.

New sensitive and adequately specific noninvasive tests are developing that may guide therapeutic strategies applicable to all IgAN stages. Multivariable pathophysiological processes may mediate IgAN initiation and progression, although IgAN is attributable to mesangial IgA or IgA immune complex (IC) deposition. The nephritogenic roles of galactose-deficient IgA1 (Gd-IgA1) and Gd-IgA1 bound with anti-glycan IgG in an IC (IgA/IgG-IC) have been discussed [16–20]. Berthoux et al. [21] recently reported that Gd-IgA1 and IgA/IgG-IC may have a predictive value for outcome of renal death in IgAN. We examined these biomarkers from a perspective that is different from their study. The present study examined whether serum levels of these noninvasive biomarkers can be a potential index for the disease activity of IgAN equivalent to urinalysis, in patients with complete or partial clinical remission after steroid pulse therapy in combination with tonsillectomy (TSP) whose clinical data and serum were obtained 3–5 years after TSP.

## Materials and methods

### Patients and treatment

IgAN diagnosis requires renal biopsy with IgA as the dominant or co-dominant Igs in a typical mesangial distribution in the absence of clinical and laboratory evidence of systemic disease. We enrolled IgAN patients showing complete/partial clinical remission after TSP from 1999−2001 in Sendai Shakaihoken Hospital and who could be followed up and whose serum could be obtained serially for 3–5 years after TSP. Clinical remission was defined as negative proteinuria and hematuria as assessed using a dipstick test and/or a urinary erythrocyte count of <5 cells per high-power field during 3 consecutive visits. We defined patients with complete remission as those who showed no further urinary abnormalities throughout the observation period after urinary abnormalities disappeared. Patients who exhibited a relapse of proteinuria and/or hematuria after remission were excluded from the complete remission group, but were included in a partial remission group.

The steroid pulse therapy included 0.5 g methylprednisolone per day for 3 consecutive days, 3 times a week, for at least 1 week after tonsillectomy. Furthermore, 0.5 mg/body weight (kg) prednisolone was administrated once every 2 days for 6–12 months with a gradual tapering of the dose within 1 year [22]. Patients who had received a kidney transplant or who required dialysis were excluded from this study. This study was approved by the ethics committee of the Sendai Shakaihoken Hospital at Miyagi, Japan, and all patients provided written informed consent.

### Clinical, laboratory and pathological data

We collected the baseline clinical data immediately before TSP, while qualitative hematuria and proteinuria data and serum were collected at a minimum of three time points, i.e., immediately before, 1 year after, and 3–5 years after TSP. Baseline clinical data (age, sex, duration from onset to tonsillectomy, systolic blood pressure, total protein, albumin, blood urea nitrogen, serum creatinine, creatine clearance rate [CCr], quantitative proteinuria, amount of proteinuria, and quantitative hematuria) and histological findings were collected from hospital medical records. CCr was calculated based on the mean 24-h urine collection and adjusted for body surface area. Hematuria was evaluated by both dipstick and microscopy in about 70 % of evaluation points, while dipstick evaluation was carried out in all points. Since there was clear correlation in hematuria between both methods, we used quantitative data by dipstick analysis for this study. The histological findings were evaluated based on the index of the glomerular lesion (IGL), as previously reported [23]. IGL is a histological score which is graded from 0−4 with a modification to evaluate sclerotic changes.

### Measurement of serum Ig, Gd-IgA1 and IgA/IgG-IC by ELISA

We measured serum Ig, Gd-IgA1, and IgA/IgG-IC at the same time, with all stock serum samples taken immediately before, 1 year after, and 3–5 years after TSP.

Serum IgA and IgG levels were determined using capture ELISA [17, 24]. ELISA plates were coated with 1 μg/ml of the F(ab’)_2_ fragment of goat IgA specific for human IgA and IgG (Jackson Immuno Research Laboratories Inc., West Grove, PA, USA). The captured Igs were then detected using a biotin-labeled F(ab’)_2_ fragment of goat IgG anti-human IgA, or IgG antibody (BioSource). Avidin-conjugated horseradish peroxidase (ExtrAvidin; Sigma-Aldrich) and peroxidase chromogenic substrate *o*-phenylenediamine/H_2_O_2_ (Sigma-Aldrich) were then added. The color reaction was stopped with 1 M sulfuric acid, and the absorbance was measured at 490 nm using the EL312 BioKinetics Microplate Reader (BioTek). The results were calculated using DeltaSoft III software (BioMetallics).

High-adsorption polystyrene 96-microwell plates (Nalge Nunc International, Rochester, NY, USA) were coated overnight with 2.5 μg/ml F(ab’)_2_ fragments of goat IgG anti-human IgA (Jackson Immuno Research Laboratories) in phosphate-buffered saline (PBS). Coated plates were blocked with 2 % bovine serum albumin (BSA; Sigma-Aldrich) in PBS containing 0.05 % Tween-20 (PBST) and serial two-fold dilutions of duplicate samples and standards in blocking solution were incubated overnight at 4 °C. The captured IgA was subsequently desialylated by treatment for 3 h at 37 °C with 10 mU/ml neuraminidase (Roche) in 10 mM sodium acetate buffer (pH = 5). Samples were then incubated for 3 h at 37 °C with GalNAc-specific biotinylated HAA lectin (Sigma-Aldrich) diluted 1:500 in blocking buffer [16]. The bound lectin was detected with avidin-conjugated horseradish peroxidase and the reaction was developed as described above. HAA reactivity of IgA1 of each sample was calculated as the optical density (OD)/1 μg of IgA. Gd-IgA1 (Ale) purified from the plasma of a patient with IgA1 multiple myeloma was treated with neuraminidase and used as the standard [16, 18].

Serum IgA/IgG-IC was determined using cross-capture ELISA [25]. High-adsorption polystyrene 96-microwell plates were coated with 1 μg/ml F(ab’)_2_ fragments of goat anti-human IgG (Jackson Immuno Research Laboratories). After washing and blocking with 1 % BSA in PBST, samples were diluted 11-fold with the same buffer. The captured Ig was detected with a horseradish peroxidase (HRP)-labeled F(ab’)_2_ fragment of goat IgG anti-human IgA (BioSource) and the reaction was developed as described above.

### Statistical analysis

Statistical analysis was performed using Stata version 11 (StataCorp, College Station, TX, USA). Normally distributed continuous variables were expressed as the mean ± SD and compared using the Student’s *t* test. Non-normally distributed continuous variables were expressed as the median (interquartile range) and compared using the Mann–Whitney *U* test. Categorical variables were expressed as numbers (proportions) and analyzed using the chi-squared test or Fisher’s exact test. The trend for each value was analyzed using the Jonckheere−Terpstra [26] test. All probability values were 2-tailed and all confidence intervals were computed at the 95 % level.

## Results

### Patient characteristics

In this study, we enrolled 50 IgAN patients with complete or partial clinical remission after TSP. The basic characteristics of the enrolled patients (*N* = 50) whose clinical parameters could be collected are summarized in Table 1. The study population included 40 % males with a median age of 37 years. The average CCr and urinary protein excretion levels were 98.2 ml/min and 0.54 g/day, respectively. A total of 52 % of the patients had complete clinical remission after TSP. Only the duration from onset to tonsillectomy was significantly different among patients with complete or partial remission after TSP (Table 2).

**Table 1:** Clinical background of IgAN patients

	Number of patients (*N* = 50)
Age	37 (25–48)
Sex (male %)	20 (40.0 %)
Onset to tonsillectomy (years)	2.0 (1.0–4.0)
SBP (mmHg)	122.3 ± 20.5
TP (g/dl)	6.8 ± 0.57
Albumin (g/dl)	4.2 ± 0.41
BUN (mg/dl)	15 ± 5.8
S-Cre (mg/dl)	0.82 ± 0.34
CCr (ml/min)	98.2 ± 26.8
UP (dipstick)	3+; 13, 2+; 8, 1+; 19, ± or −: 10
UP (g/day)	0.54 (0.3–1.3)
U-OB (dipstick)	3+; 27, 2+; 17,1+; 4, ±; 2
IGL score	1.47 (1.3–1.99)
Gd-IgA1 (units/mg IgA)	117.3 ± 45.6
IgA/IgG-IC (OD)	0.81 ± 0.31

Continuous data are presented mean ± SD or median [IQR], and categorical data as number of patients (%)

*SBP* systolic blood pressure, *BUN* blood urea nitrogen, *S-Cre* serum creatinine, *CCr* creatinine clearance, *UP* urinary protein, *U-OB* urinary occult blood, *IGL* index of the glomerular lesion, *TP* total protein

**Table 2:** Clinical background and course of complete and partial remission groups

	Complete remission (*N* = 26)	Partial remission (*N* = 24)	*P*
Age	32.0 (24–43)	40.5 (28.5–50)	0.13
Sex (male %)	13 (50 %)	7 (29.2 %)	0.13
Onset to tonsillectomy (years)	1.0 (1.0–3.0)	3.0 (2.0–4.0)	0.02
SBP (mmHg)	122.4 ± 20.2	123.5 ± 21.4	0.85
TP (g/dl)	6.8 ± 0.51	6.8 ± 0.64	0.7
Albumin (g/dl)	4.3 ± 0.36	4.1 ± 0.44	0.13
BUN (mg/dl)	13.8 ± 3.7	16.1 ± 7.4	0.18
CCr (ml/min)	103.3 ± 24.2	92.8 ± 28.8	0.06
UP (g/day)	0.45 (0.3–1.0)	0.75 (0.36–1.45)	0.19
IGL score	1.40 (1.29–1.79)	1.62 (1.35–2.2)	0.18
S-Cre (mg/dl)			
Baseline	0.77 ± 0.19	0.82 ± 0.41	0.87
1 year	0.78 ± 0.24	0.84 ± 0.43	0.56
3–5 year	0.77 ± 0.26	0.91 ± 0.70	0.34
UP (dipstick)			
Baseline	3+; 7, 2+; 2, 1+; 9, ±or −; 8	3+; 6, 2+; 6, 1+; 10, ± or −; 2	0.17
1 year	2+; 1, 1+; 6, ± or −; 19	2+; 6, 1+; 7, ± or −; 11	0.01
3–5 year	± or −; 26	3+; 1, 2+; 6, 1+; 7, ± or −; 10	<0.001
U-OB (dipstick)			
Baseline	3+; 11, 2+; 13, 1+; 1, ±or −; 1	3+; 16, 2+; 4, 1+; 3, ± or −; 1	0.23
1 year	3+; 1, 2+; 2, 1+; 2, ± or −; 21	3+; 3, 2+; 1, 1+; 9, ± or −; 11	0.01
3–5 year	± or −; 26	3+; 2, 2+; 4, 1+; 8, ± or −; 10	<0.001

Continuous data are presented mean ± SD or median [IQR], and categorical data as number of patients (%). *P* based on complete remission and partial remission comparison

*SBP* systolic blood pressure, *BUN* blood urea nitrogen, *S-Cre* serum creatinine, *CCr* creatinine clearance, *UP* urinary protein, *U-OB* urinary occult blood, *IGL* index of the glomerular lesion, *TP* total protein

### Cross-sectional analysis

**Fig. 1:**
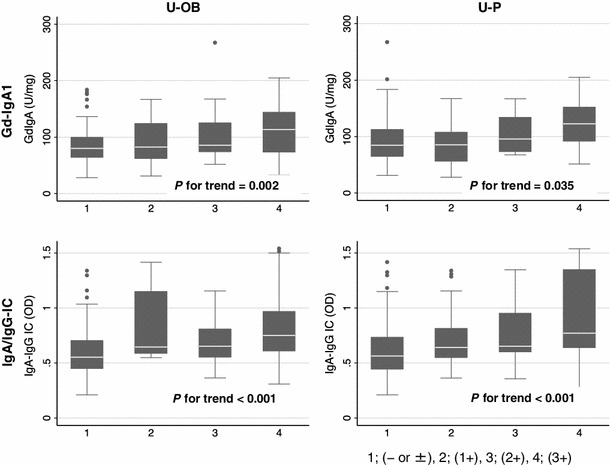
Cross-sectional analysis of the correlation between severity of hematuria/proteinuria and serum Gd-IgA1 or IgA/IgG-IC levels. Significant correlations were found between serum Gd-IgA1 levels and hematuria (U-OB) and proteinuria (U-P), as determined by dipstick tests. Furthermore, significant correlations were also detected between serum IgA/IgG-IC levels and severity of urinary findings [1; (− or ±), 2; (1+), 3; (2+), 4; (3+) on *x* axis]

We first performed cross-sectional analysis to evaluate potential correlation between severity of hematuria or proteinuria and serum levels of Gd-IgA1 or IgA/IgG-IC (Fig. 1). Significant correlations were observed for serum Gd-IgA1 levels and severity of hematuria (*P* for trend = 0.002) and proteinuria (*P* for trend = 0.035). Furthermore, significant correlations were observed for IgA/IgG-IC levels and severity of urinary findings (hematuria; *P* for trend <0.001, proteinuria; *P* for trend <0.001).

### Longitudinal analysis of patients with hematuria

**Fig. 2:**
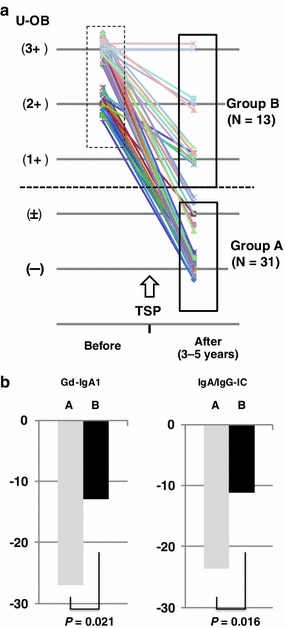
Longitudinal analysis of patients with hematuria. Forty-four patients with heavy hematuria of >2+ in dipstick tests before TSP were divided into group A, which contained 31 patients with complete remission of hematuria, and group B, which contained the remaining patients who retained hematuria, during the 3–5-year follow-up period (**a**). Group A patients had a significantly higher percentage decrease in both serum Gd-IgA1 (*P* = 0.021) and IgA/IgG-IC (*P* = 0.016) levels than group B patients (**b**)

We divided the 44 patients (91.7 %) with heavy hematuria of >2+ by dipstick before TSP into group A [31 patients (64.6 %) with complete remission of hematuria] and group B (remaining patients who retained hematuria during the 3–5-year follow-up period) (Fig. 2a). There was no significant difference in serum Gd-IgA1 and IgA/IgG-IC levels before TSP in both groups [group A vs B, Gd-IgA1 (U/mg IgA); 122.1 ± 48.0 vs 107.7 ± 43.0, *P* = 0.36, IgA/IgG-IC (OD); 0.77 ± 0.31 vs 0.85 ± 0.29, *P* = 0.43]. Group A patients had a significantly higher percentage decrease in Gd-IgA1 (*P* = 0.021) and IgA/IgG-IC (*P* = 0.016) serum levels after TSP than group B patients (Fig. 2b).

### Longitudinal analysis of patients with proteinuria

**Fig. 3:**
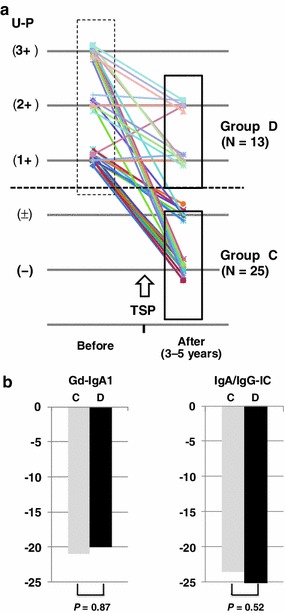
Longitudinal analysis of patients with proteinuria. Thirty-eight patients with proteinuria before TSP were divided into groups C and D, with or without proteinuria 3–5 years after TSP (**a**). Cross-sectional analysis revealed significant correlations between severity of proteinuria and serum Gd-IgA1 and IgA/IgG-IC levels, but the percentage decrease in serum Gd-IgA1 and IgA/IgG-IC levels did not differ between the groups (**b**)

We then divided the 38 patients (79.2 %) with proteinuria before TSP into groups C (*N* = 25) and D (*N* = 13), with or without proteinuria 3–5 years after TSP, respectively (Fig. 3a). There was a significant difference in serum Gd-IgA1 levels, but not in IgA/IgG-IC levels, before TSP in both groups [group C vs D, Gd-IgA1 (U/mg IgA); 102.2 ± 37.6 vs 133.3 ± 41.4, *P* = 0.03, IgA/IgG-IC (OD); 0.81 ± 0.30 vs 0.98 ± 0.33, *P* = 0.11). Cross-sectional analysis indicated significant correlations between proteinuria severity and serum Gd-IgA1 and IgA/IgG-IC levels. However, the percentage decreases in Gd-IgA1 (*P* = 0.87) and IgA/IgG-IC (*P* = 0.52) serum levels after TSP were not significantly different between the 2 groups (Fig. 3b).

The average percentage decrease in IgA/IgG-IC levels before and after 3–5 years was 20 ± 17 in all patients. Next, we divided the patients according to the average percentage decrease in IgA/IgG-IC serum levels before TSP and 3–5 years after TSP into large delta IC (>20) and small delta IC (≤20) groups, and analyzed laboratory data for the patients in the large delta IC group. In this large delta IC group (*N* = 25; 50 %) of patients who had a greater than average percentage decrease (>20) in IgA/IgG-IC serum levels, proteinuria after 3–5 years was persistent only in 4 patients (16 %) who had severe sclerotic glomerular lesions before TSP (data not shown).

## Discussion

This is the first report to demonstrate that assessment of IgAN activity based on urinary abnormality correlates with changes in serum levels of Gd-IgA1 and IgA/IgG-IC. This study indicates that Gd-IgA1 and IgA/IgG-IC could be extremely useful components for evaluation of IgAN activity in a noninvasive manner.

Annual routine screening for urinary abnormalities is conducted in school-aged children to adults in Japan [12, 13], and these screening procedures markedly increase the percentage of early stage IgAN patients presenting with microscopic hematuria and the overall IgAN prevalence. Indeed, chance microscopic hematuria is a leading event for renal biopsy in Japan [5, 7–10, 12, 13]. This observation suggests that hematuria is an initial manifestation of early stage IgAN and a primary manifestation of active IgAN. Recent studies revealed abnormalities of IgA1 glycosylation and formation of autoantibodies to these aberrantly glycosylated IgA1 molecules as key factors in the pathogenesis of IgAN [17–20]. Excessive production of IgA1 is an unlikely sole cause of glomerular IgA, because in IgA myeloma patients IgA rarely deposits in the kidney. Furthermore, only approximately one-third to a half of IgAN patients have increased IgA levels [1, 27, 28]. Thus, a structurally, immunologically, or physicochemically abnormal IgA1 molecule, such as Gd-IgA1, produced by IgAN patients, has been considered as a possible cause of glomerular IgA deposition. Indeed, serum Gd-IgA1 levels are elevated in IgAN patients where they are mainly regulated by genetic and environmental factors [16, 20, 29]. However, the clinical association between Gd-IgA1 levels and their clinical manifestation has not been completely evaluated. It is notable that serum Gd-IgA1 levels correlated with severity of hematuria. In addition, the disappearance or improvement of hematuria after TSP correlated with a decrease in serum Gd-IgA1 levels. These findings indicate that formation of Gd-IgA1 and Gd-IgA1-containing IC are key steps in the pathogenesis of IgAN, leading to glomerular deposition of these complexes and development of glomerular injury with subsequent hematuria [20]. However, specific serum Gd-IgA1 levels were still detected, even in patients who experienced complete remission after TSP. The absolute amounts of serum Gd-IgA1 were also independent of severity of hematuria before TSP. Therefore, threshold levels of Gd-IgA1 that induce hematuria may differ among individuals. Notably, elevated levels of Gd-IgA1 have been reported also in healthy relatives of IgAN patients [29], suggesting heterogeneity of Gd-IgA1 itself for the induction of glomerular damages.

The production site of nephritogenic Gd-IgA1 remains unclear, although there are some emerging clues. For example, we noted that hematuria in some IgAN patients improved after tonsillectomy alone and this improvement was associated with decreased serum Gd-IgA1 levels (Suzuki Y et al., unpublished data). We previously reported on an animal model of IgAN in which the mucosal activation of Toll-like receptor 9 (TLR9) was involved in IgAN pathogenesis [30, 31]. Furthermore, we reported that a single nucleotide polymorphism of TLR9 was linked with IgAN progression in humans [30]. Another recent study demonstrated that IgAN patients whose serum IgA levels decreased to more than average after tonsillectomy alone (large ΔIgA) showed a significantly higher mRNA expression of TLR9 in the tonsils than IgAN patients with a smaller decrease (small ΔIgA) in these levels [32]. These findings suggest that nephritogenic Gd-IgA1 may be produced in the tonsils and that this production may involve TLR9 activation [33]. This conclusion is consistent with the observation that tonsillar TLR9 expression was elevated in IgAN patients whose serum Gd-IgA1 levels decreased significantly after tonsillectomy alone (Suzuki Y et al., unpublished data).

Increased IgA-IC levels were found in a large number of IgAN patients [27, 34]. A significant number of IgAN patients have an IC that contains both IgA1 and IgG [19, 35]. Mixed complexes with different Ig isotypes may emerge, at least in part, from specific Ig–anti-Ig interactions. Such an interaction could partly be the result of idiotype–anti-idiotype recognition, the presence of IgA rheumatoid factor (an IgA autoantibody specific to the Fc region of IgG), or IgG–anti-IgA as well as IgA1 anti-glycan antibodies [24]. Indeed, idiotype-positive antibody levels correlated with the clinical status of IgAN patients, as defined by their urinary abnormalities [36]. Recently, it was suggested that IgAN is characterized by a circulating IC composed of Gd-IgA1 and a glycan-specific IgG antibody. Suzuki et al. [18] reported that serum glycan-specific IgG antibody levels could differentiate between IgAN patients and healthy or diseased controls, with 88 % specificity and 95 % sensitivity. In addition, increased levels of this antibody in sera of IgAN patients correlated well with proteinuria. This study evaluated serum IgA/IgG-IC levels, and our findings regarding proteinuria and IgA/IgG-IC levels are consistent with previous studies [18, 35]. *O*-linked carbohydrates in the hinge region of IgA1 considerably affect IgA1 reactivity with such glycan-specific autoantibodies, and the subsequent IC formation may incite glomerular damage, leading to proteinuria and hematuria [18]. Gharavi et al. [29] reported that blood relatives of IgAN patients had increased serum Gd-IgA1 levels even in the absence of nephropathy, suggesting that additional events may be required for complete IgAN progression. Thus, IC formation with Gd-IgA1 and glycan-specific IgG antibody may be one of the second ‘hit’ events [18, 20]. It is generally known that higher molecular ICs have a higher phlogogenic capacity via the activation of Fc receptors [37]; hence, serum IgA/IgG-IC levels may correlate with severity of glomerular damage leading to proteinuria better than Gd-IgA1 alone. These facts are consistent with present findings in a cross-sectional analysis that serum levels of IgA/IgG-IC were more correlated with severity of urinary abnormalities than those of Gd-IgA1.

In conclusion, we showed in this study that disease activity assessment by hematuria and proteinuria correlated with changes in serum Gd-IgA1 and IgA/IgG-IC levels in most IgAN patients, providing novel value for these new noninvasive and real-time disease activity markers. Although further validation with a larger cohort will be required, clinical application, such as IgAN activity score or risk score, with these markers as principal components could be extremely useful for guiding the therapeutic approaches applicable in all stages of IgAN.
